# Were medicine quality and pharmaceutical management contributing factors in diminishing artemisinin efficacy in Guyana and Suriname?

**DOI:** 10.1186/1475-2875-13-77

**Published:** 2014-03-03

**Authors:** Victor S Pribluda, Lawrence Evans, Edgar Barillas, John Marmion, Patrick Lukulay, Jaime Chang

**Affiliations:** 1 Promoting the Quality of Medicines Program (PQM), Global Health Impact Programs (GHIP), United States Pharmacopeial Convention (USP), 12601 Twinbrook Parkway, Rockville, MD 20852-1790, USA; 2 Systems for Improved Access to Pharmaceuticals and Services (SIAPS), Center for Pharmaceutical Management, Management Sciences for Health (MSH), 4301 N Fairfax Dr Suite 400, Arlington, VA 22203, USA; 3 Office of Health and Education, United States Agency for International Development (USAID)/Peru, Avenida La Encalada cdra 17 s/n, Surco, Lima 33, Peru

## Abstract

**Background:**

Recent studies in Guyana and Suriname unveiled diminished efficacy of artemisinin derivatives based on day-3 parasitaemia. The migrant characteristics of the population at risk and the potential development of resistance pose a serious health threat in the region. Assessment of factors that may have contributed to this situation is warranted, and analysis of the data generated in those countries on quality and pharmaceutical managements of anti-malarials may contribute to a better understanding of this occurrence.

**Methods:**

Data on malaria medicine quality and pharmaceutical management, generated in the context of the Amazon Malaria Initiative (AMI), was reviewed and discussed.

**Results:**

Numerous substandard artemisinin-containing malaria medicines were identified in both countries, particularly in Guyana, where a larger number and variety of anti-malarials were sampled. Poor quality was more frequent in the private and informal sector than in the public sector, posing a greater threat to the populations at risk, which are mostly located in hard to reach areas with scarce public facilities. Stock-outs identified in the public sector in Guyana could enhance the need to access those alternative sectors, exacerbating the risk of utilizing poor quality medicines. The availability of monotherapies and other non-recommended therapies for *Plasmodium falciparum* malaria, could also have contributed to the diminished efficacy. The type of quality deficiencies identified -reduced content of active pharmaceutical ingredient (API) and/or poor dissolution- and the irrational use of non-recommended treatments could result in non-sustained or lower levels of API in blood, favouring survival of more resistant mutants by exposing parasites to sub-lethal doses of the active ingredient.

**Conclusions:**

The quality of malaria medicines and the availability and use of non-recommended treatments could have played a role in the diminished efficacy of artemisinin derivatives described in Guyana and Suriname. However, also other factors need to be considered and a more comprehensive and extensive assessment on quality and pharmaceutical management is necessary to establish a tighter cause-effect correlation. Nevertheless, relevant authorities in these and neighbouring countries should take into consideration the reviewed data to properly address the problem when implementing corrective actions.

## Background

Artemisinin derivative resistance was reported initially in Southeast Asia [1,2], and subsequently the World Health Organization (WHO) developed two important documents addressing global reports on artemisinin resistance and resistance containment plans [3,4]. Two recent anti-malarial drug efficacy studies performed with artemether-lumefantrine combinations in Guyana and Suriname show higher parasitaemia levels at day 3 than those encountered in previous years [5,6]. Although these data were not considered definitive evidence of artemisinin resistance and did not merit the designation of these as tier 1 countries, it was pointed out that resistance in Southeast Asia showed initially a similar trend [7]. Updated information on the status of malaria in both countries is available in a recent WHO report [8]. In regards to the population at risk for *Plasmodium falciparum*, it is mostly constituted of migrant gold miners in both countries, as it has been documented in a recent review [9] for Suriname and in a publication that highlighted the risk of emergence of resistance to artemisinin derivatives in French Guyana [10]. As indicated in [11], similar populations at risk are found in Guyana, including the west border neighboring with Venezuela and Brazil. Taking into consideration the migrant characteristics of the population at risk, if resistance appears, it may rapidly spread into neighboring countries and beyond.

These findings in Guyana and Suriname prompt the urgent need to perform a thorough analysis of the conditions and behaviours that may have contributed to this occurrence, which could result in the impairment of *P. falciparum* malaria therapy. The regional and global consequences of resistance to artemisinin derivatives cannot be overstated, particularly because, as of today, there is no effective comparable alternative.

Management Sciences for Health (MSH) and the United States Pharmacopeial Convention (USP) have been involved in the Amazon Malaria Initiative (AMI) since the early 2000s, through collaborative agreements established with the United States Agency for International Development (USAID). The 'Systems for Improved Access to Pharmaceuticals and Services’ (SIAPS/MSH) and the 'Promoting the Quality of Medicines’ (PQM/USP) programmes provide technical assistance to improve countries’ pharmaceutical management and their quality assurance and quality control systems.

Poor quality medicines, irrational use, and/or lack of adherence to treatment regimens could be factors contributing to the onset of microbial resistance. Therefore, it was considered appropriate to re-examine relevant information reported previously under AMI by PQM and SIAPS for Guyana and Suriname [11-14] to see whether it hints at factors that could have contributed to the efficacy results reported subsequently in the region. The inclusion of Brazil information is warranted because the three countries share endemic border areas with large mobile mine-working populations.

## Methods

Publicly available information, mostly generated through PQM and SIAPS activities in AMI, was reviewed and assessed for information on malaria medicine’s quality and pharmaceutical management. Additional data for review was gathered through literature searches at PubMed.

## Results

### Assessment of medicine’s quality information

Though the quality studies shed light on availability of non-recommended treatments and uncovered extensive commercialization of anti-malarials in the private and informal market, this article will focus only on available quality data for medicines containing artemisinin derivatives collected in the public, private and informal sectors in Guyana, Suriname and Brazil. It is of note that the first-line treatment for *P. falciparum* malaria in Guyana and Suriname is the fixed dose combination (FDC) of artemeter-lumefantrine, while Brazil utilizes also an artesunate-mefloquine FDC [8].

### Quality of anti-malarials in the public sector in Brazil and Guyana

For the purpose of performing quality control, AMI countries collected anti-malarial medicines between 2005 and 2010. The results have been reported in [12], which also contains detailed information on the medicines collected and the sampling methodology, as well as failures percentages by country and type of medicine. Most of the medicines collected in Brazil (301/313 between 2006 and 2008) and Guyana (220/289 between 2007 and 2010) were sampled in the public sector. Although the report provides information on the results obtained with screening tests in the field, in Brazil and Guyana –following protocol– failed samples and a subset of passing samples were reanalyzed for certain tests at the Official Medicine Control Laboratory (OMCL). In the majority of the cases, there was concordance between the field tests and those repeated at the OMCL (see table four in [12]). Artemisinin derivatives comprised four of the anti-malarials collected in Brazil and 92 of those collected in Guyana. In the case of Suriname, 20 anti-malarials were collected in 2007; 15 were artemisinin-containing medicines. Because samples in Suriname were collected in the informal sector, results will be discussed in the following section.

In Brazil and Guyana, medicines were visually inspected and assessed for disintegration, identity, content, and impurities (the three latter through thin layer chromatography (TLC)). These are tests that require minimal equipment and can be performed in the field. The percentage of anti-malarials that failed quality control tests in Brazil and Guyana were 6.4 and 7.3%, respectively, of which disintegration and TLC failures accounted for 4.5% in each country. However, no failures were reported in disintegration or TLC for artemisinin derivatives. The only type of failure found in artemisinin-containing medicines in both countries was that some samples were beyond their expiry date. For all anti-malarial medicines tested in Brazil (313), six (1.9%) were expired, among the latter only one artesunate. In the case of Guyana, from 289 samples, 8 (2.8%) were expired, and among the latter three artemether and three FDC of artemether and lumefantrine. Based on the results discussed in this section, the quality of anti-malarials in the public sector in Brazil and Guyana apparently was not a major risk factor for lesser artemisinin efficacy.

### Quality of anti-malarials in the private and informal sector in Guyana and Suriname

For information on the medicines collected (Active Pharmaceutical Ingredient-API, and sector) and the sampling methodology utilized in the Guyana and Suriname study see [11]. The samples were analyzed at laboratories operating in compliance with internationally recognized standards (Peru OMCL: ISO:EIC 17025:2005 accreditation and WHO prequalification, and USP: ISO:EIC 17025:2005 accreditation). All collected medicines were analysed through the comprehensive battery of validated methodologies detailed in [11], not the field tests utilized in the studies described in the section above. This allowed for better accuracy in the measurement of content and impurities and also for the assessment of additional quality attributes such as dissolution and uniformity of content. As discussed below, these sectors are the ones more readily available to the migratory mining population, which constituted the majority of malaria patients assessed during the efficacy studies that showed higher parasitaemia after three days of treatment with an artemisinin derivative.

### Guyana

Malaria medicines were sampled in the private and informal sectors in 2009. From these, 42% (32/77) were collected in the private sector and 58% (45/77) in the informal sector. In total, 46 samples (60%) were collected in the three high malaria incidence regions, while 31 samples (40%) were from three non-endemic 'source’ regions; the latter being regions with urban centres where people purchase medicine before travelling to the more remote endemic areas. Therefore, medicines from both sectors and types of regions were more or less equally represented in this study. Most of the samples (64%; 40/77) contained artemisinin derivatives, of which 7.5% (3/40) were monotherapy (artesunate).

The artemisinin-containing medicines collected included a monotherapy (Artesunate) and fixed-dose combinations (Artecom: dihydroartemisinin, piperaquine, trimethoprim, primaquine; Artemos-40: dihydroartemisinin, piperaquine, trimethoprim; Co-Ariante: artesunate, sulphamethoxypyrazine, pyrimethamine; Artemos-Plus: artemether, lumefantrine) containing artemisinin derivatives. Except for Artemos-Plus, none of the other artemisinin-containing medicines collected were included in the anti-malarial treatment policy of the country, and Artecom®, prevalent among all anti-malarials collected (26/77), is not even registered in Guyana. A fixed-dose combination of dihydroartemisinin, piperaquine and trimethoprim was also a prevalent medicine found and collected (34%; 26/77).

From all the medicines sampled in the private and informal market, 58% (45/77) failed quality control testing, which is a significantly higher percentage than the 7.3% discussed above for the public sector. Two factors contributed, in part, to this larger number: (a) All Artecom samples (26) failed visual inspection because of incomplete information on the primaquine tablet content in the insert, and (b) Failures in Uniformity of Dosage Units, some impurities and dissolution are not assessed with the field screening tests utilized in [12]. However, even if from the total of 45 failures, that could have been detected with the a field screening test (TLC), the percentage failing would be 17% (13/77), still significantly higher than the 4.5% (13/289) reported for medicines collected mostly in the public sector [12]. Thus, one may conclude that the anti-malarials commercialized in the private and the informal sectors represent a much higher risk for the population than those provided free of charge at public sector facilities.

More important in the context of potential onset of resistance to artemisinin, is that 67% (33/45) of the failures identified in the private and informal sector were in artemisinin-containing medicines. Some of those failures, such as content and dissolution, could result in diminished levels of active pharmaceutical ingredients in the blood stream. All the samples collected from four out of five different artemisinin-containing medicines failed. Although no further investigation was done on the artemisinin-containing medicines that failed, based on the deviations from the declared content or their claimed source, none of them could be considered, *prima facie*, counterfeits. These results show an additional threat that malaria patients face in endemic mining areas, where private and informal facilities are prevalent and public sector facilities may be difficult to access.

For all anti-malarials, the risk of purchasing poor quality medicines existed both in the private sector (34%; 11/32) and the informal sector (16%; 7/45), and were equally prevalent in endemic regions (24%; 11/46) and non-endemic source regions (23%; 7/31). In endemic regions, almost twice as many samples (seven) failed in the informal sector than in the private sector (four), and in non-endemic source regions, all failures (seven) were identified in the private sector. Thus, based on these findings there is a clear risk of purchasing poor quality malaria medicines in the private or informal sector, both in endemic areas as well as non-endemic regions.

### Suriname

The results reported in [12] in the informal sector showed no failures, though this data should be taken cautiously. Of the 15 anti-malarials containing an artemisinin derivative, 12 had dihydroartemisinin, one as a monotherapy and 11 as the fixed-dose combinations dihydroartemisinin, piperaquine and trimethoprim; for those, only disintegration was assessed because the field test for TLC was not available at the time. In addition, the paucity of samples in Suriname (20 in 2007) precludes making any generalization for this country based on that sole report.

In Suriname, medicines were collected during 2009 only in facilities from the informal sector [11], mostly along rivers that constitute the supply-line in mining areas; some samples were collected also in the capital, Paramaribo City. The prevalence of Artecom® in this sector was also very high, constituting 86% (49/57) of all sampled medicines, and those were analysed at USP.

Of note is that three of the eight lots of Artecom® sampled in Suriname had the same lot number as those sampled in Guyana. The existence of the same lots in the two countries would suggest that there is commerce of medicine across borders and/or that supplier(s) of this unregistered medicine work across borders. All Artecom® samples failed visual inspection because they lacked information on the content of the co-packaged primaquine tablet, though the co-packaged fixed-dose combination tablets passed analytical testing.

Based on these assessments, quality does not seem to be a risk factor for the most prevalent medicine collected. However, as discussed below, the risk posed by this extensively utilized non-registered medicine is related to irrational use and lack of adherence to treatment guidelines.

### Assessment of pharmaceutical management information

#### Supply of anti-malarials in the public sector

As reported in the June 2013 issue of the quarterly regional monitoring of anti-malarials bulletin, 56% of medicines normally found in the central warehouse of Guyana was stocked out [13]. Supply issues, particularly in Guyana, could create a discontinuous supply of medicines at the point of delivery and encourage patients to access these medicines in the more risky private and informal sectors.

#### Availability of anti-malarials in the private and informal sector

In AMI countries, free treatment is provided to patients by the Ministries of Health. However, as clearly uncovered by the quality studies discussed above, there is ample commercialization of anti-malarials beyond the public sector. The risk posed by that is reflected not only in the quality of those products, but in the access to medicines that are not registered in the countries and for which there is no evidence of being efficacious. In addition to the improper use of those products, there was evidence of availability of artemisinin-derivatives monotherapy, whose use is against WHO guidelines.

Commercialization of anti-malarials is also well documented in Guyana in an anti-malarials availability assessment performed in the public and private sector from several AMI countries [14]. More than 90% of the number of units of anti-malarials distributed in the private sector contained artemisinin derivatives that were not registered in the country.

It is worth noting that in terms of the availability and use of anti-malarials in these sectors, that similar behaviours and environment are described for the population at risk in Suriname [9] and French Guyana [10] and most probably also applies to Guyana.

## Conclusions

The evidence available for Guyana and Suriname identifies several risk factors that individually, and even more collectively, may have had a role in the reduced efficacy of artemisinin derivatives recently found in these two countries. A high percentage of anti-malarials collected in Guyana had quality problems. Furthermore, in both countries, non-recommended treatments including artemisinin monotherapy and non-registered artemisinin derivatives were readily available beyond the public sector, in areas where patients and travellers are more likely to use the private and informal sector to buy these medicines.

Although only mefloquine and quinine samples were identified that had no activity ingredient at all, several artemisinin derivative-containing medicines had content and/or dissolution problems. These type of failures could result in lower than required therapeutic levels in patients’ blood, and dosage below the optimal level will kill the susceptible parasites while the drug-resistant parasites survive and contribute to their spread [4]. Of note is that the poor quality anti-malarials were found mostly in the private and informal sectors, which are prevalent and easily accessible to patients in endemic areas.

In addition to quality problems, these studies identified artemisinin-derivative monotherapy and numerous unregistered artemisinin-based combination therapies (ACT). The former could cause higher survival rates of the less sensitive forms of the parasite due to their rapid clearance from the blood. Regarding the latter, even assuming that the non-registered ACT would be efficacious (which has to be proven for this population), lack of guidance for proper use by patients and lack of adherence to treatment would most probably be associated with the use of those medicines for shorter times than required (when febrile episodes subside), which in turn would result in incomplete elimination of the parasite and selective survival of resistant strains. Finally, the presence of the same lots of an unregistered anti-malarial in these countries is consistent with porous borders, as is the case for the movement of migrant workers. Both factors may be associated and potentiate the risks of spreading of the parasites less sensitive to artemisinin.

One has to be cautious, however, when interpreting the quality data reviewed here, and it is important to consider, amongst other, the following factors: the sample size of these studies was not very large; sampling size and sampling methodologies could affect the results (failure percentages); not all relevant geographic areas were covered; some of the data discussed in this article is not very recent. Regarding the latter, the authors are not aware of any recent quality monitoring activity performed in risk areas in neither of these two countries. It is also important to highlight that other factors not considered in this article may have had a significant impact on the efficacy of the treatment, particularly behaviour patterns in population at risk such as migrant workers travelling across international borders. In addition, these are hard to reach geographical areas poorly covered by public health services, resulting in treatment and patients outreach far from optimal. Some of these problems are discussed in [9,10], especially for Suriname where diminished efficacy has been observed despite an otherwise effective and comprehensive approach to reach target populations and combat malaria [9].

To conclude, the evidence and information for the risk factors assessed here have been in the public domain for years. Now that higher parasitaemia after three days of treatment has been uncovered and the risk of resistance unveiled, it is expected that health authorities, individually and collectively, will intensify national and regional efforts to address the potential contributing factors described in this brief within the context of actions taken to avoid the onset and prevent the spread of resistance.

## Abbreviations

AMI: Amazon malaria initiative; API: Active pharmaceutical ingredient; ATC: Artemisinin-based combination therapy; FDC: Fix dose combination; MSH: Management sciences for health; OMCL: Official medicine control laboratory; PQM: Promoting the quality of medicines; SIAPS: Systems for improved access to pharmaceuticals and services; TLC: Thin layer chromatography; USAID: United states agency for international development; USP: United states pharmacopeial convention; WHO: World health organization.
